# Telemedicine in Psychiatry: benefits and challenges of the the Home-Hospital Care system (COD20) project

**DOI:** 10.1192/j.eurpsy.2023.1115

**Published:** 2023-07-19

**Authors:** M. Palazzo, T. G. Prodi, M. Cerioli, D. Conti, A. Galbassini, G. Nicolini, B. Dell’Osso

**Affiliations:** 1 Department of Mental Health and Addiction, ASST FBF Sacco, Milan, Italy; 2 Department of Mental Health, Department of Biomedical and Clinical Sciences Luigi Sacco, University of Milan, ASST FBF-Sacco, Milano, Italy

## Abstract

**Introduction:**

Telepsychiatry (TP) is the use of telecommunication technologies to provide psychiatric assessment, diagnosis, treatment, and consultation. During the COVID-19 outbreak, TP has shown potential for connecting with people unable to access traditional in-person services, and also enabling patients to receive mental health care safely from home. The Home Hospital care system (Cure Ospedaliere Domiciliari; COD20) is a video consultation service developed by the University of Milan.

**Objectives:**

We aimed at investigating the potential of Telemedicine (TM) in a sample of psychiatric patients.

**Methods:**

As of now, 208 consecutive patients of an outpatient clinic belonging to ASST Fatebenefratelli-Sacco in Milan were interviewed through an online anonymous survey. Data collected were sociodemographic, job position, educational level, digital skills and both satisfaction degree and ease of use of the COD20 tool. Data were analyzed using SPSS v.27.

**Results:**

Among 208 patients, 87.7% had Internet access, 94.5% used a smartphone, 74% used a computer and 37% used a tablet. The levels of digital skills were considered intermediate-advanced in communication and information research for the majority of the cases. A high percentage of patients (80.8%) learned how to use electronic devices by themselves, while only 12.3% had an ECDL certificate. The most represented diagnoses were Mood Disorders (44.5%) and Anxiety Disorders (14%). The majority of the sample (54.8%) was visited using TM for clinical interviews: 24.7% of them used TM at least 10 times/year, and 19.8% more than 20 times/year. Among all the clinical interviews conducted using TM, 61% concerned psychiatric consultation, while 30% were dedicated to psychotherapy. The most used tool was the COD20 platform (21.9%): it was considered easy to use in 47.9% of cases, while 43.8% of patients would like to use it again in the future. The main reasons leading to the usage of TM were the difficulties in reaching the ambulatory (for 43.9%) and the workplace (for 30%).

**Image:**

**Figure:**
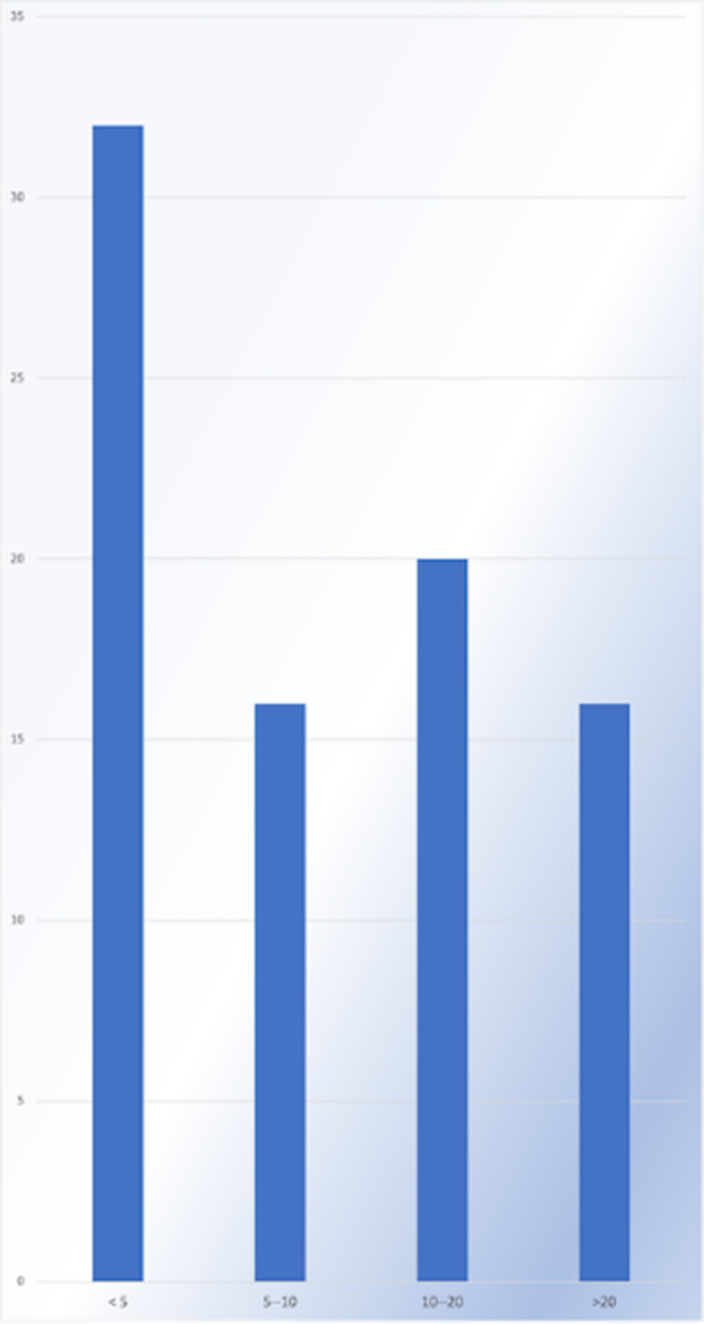

**Image 2:**

**Figure:**
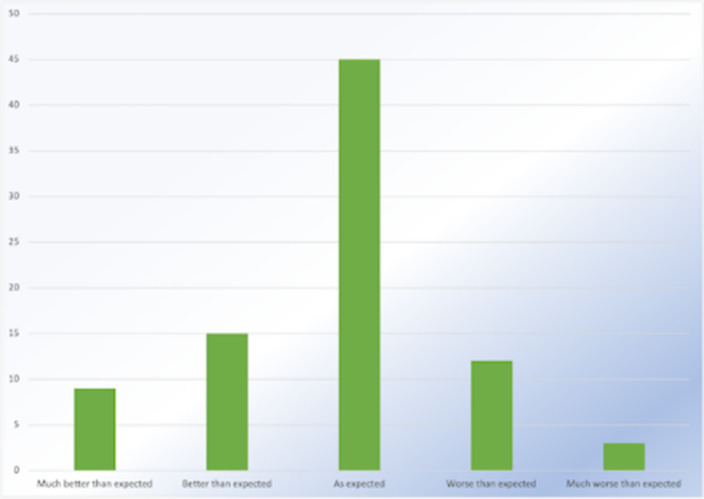

**Conclusions:**

TM represents a valid implementation in the traditional clinical practice, and we showed it is well received in terms of appreciation and ease of use. The COD20 platform could increase access to care, and overcome barriers such as distance, travel costs and time management. TP contributes to develop a more inclusive healthcare process for patients, with better performance in terms of compliance.

**Disclosure of Interest:**

None Declared

